# Supplementary material for: Cloud BioLinux: pre-configured and on-demand bioinformatics computing for the genomics community
Source: BMC Bioinformatics. 2012 Mar 19;13:42. doi: 10.1186/1471-2105-13-42 (PMC3372431; doi:10.1186/1471-2105-13-42)
Supplement: Additional file 1 — Supplementary 1 Cloud BioLinux software documentation in the form of a mini, self-contained website. Users need to download and uncompress the .zip file, and open through a web browser the "index.html" file available on the main directory. (ZIP 1823 kb). [file 1471-2105-13-42-S1.ZIP › Cloud-BioLinux-Package-Documentation/docs/lucy.html]

Bio-Linux Software Documentation Pages

Back to search form

## lucy

|  |  |
| --- | --- |
| Name | lucy |
| Description | **lucy** is a sequence cleaning utility developed at TIGR. The cleanup process carried out by **lucy** includes quality assessment, confidence reassurance, vector trimming and vector removal.  **References:**  H.-H. Chou and M.H. Holmes. DNA sequence quality trimming and vector removal. Bioinformatics, 17:12, pp. 1093-1104, 2001 |
| Homepage | http://www.tigr.org/software/ |
| Remote Documentation | http://www.complex.iastate.edu/download/Lucy2/index.html   http://www.complex.iastate.edu/download/Lucy2/tutorial/1\_guided\_1.html |
